# Case report: Pulmonary arterial hypertension in *ENG*-related hereditary hemorrhagic telangiectasia

**DOI:** 10.3389/fcvm.2022.1020762

**Published:** 2022-11-10

**Authors:** Dong Liu, Feiya Xu, Qian Gao, Zhenguo Zhai

**Affiliations:** ^1^Peking University China-Japan Friendship School of Clinical Medicine, Beijing, China; ^2^Department of Pulmonary and Critical Care Medicine, Center of Respiratory Medicine, China-Japan Friendship Hospital, National Center for Respiratory Medicine, Institute of Respiratory Medicine, Chinese Academy of Medical Sciences, National Clinical Research Center for Respiratory Diseases, Beijing, China; ^3^Department of Pulmonary and Critical Care Medicine, Capital Medical University, Beijing, China

**Keywords:** exertional dyspnea, pulmonary hypertension, hereditary hemorrhagic telangiectasia (HHT), pulmonary arterial hypertension, ENG

## Abstract

A young adult woman presented with exertional dyspnea and she had had recurrent epistaxis for more than 10 years. On physical examination, cyanosis was noted on the lips, and telangiectasias were seen on the oral mucosa and fingertips. Routine investigations revealed iron deficiency anemia and slightly elevated bilirubin. The result of right heart catheterization was indicative of pulmonary arterial hypertension (PAH). Pulmonary angiography showed arteriovenous malformations of the left upper pulmonary artery, and anterior cerebral artery malformation was seen in cranial computed tomographic angiogram. Genetic testing revealed that she and her three daughters carried heterozygous variant of ENG c.1195-1196del p.Arg399GlyfsTer2, which is characterized by pulmonary and cerebral arteriovenous malformations. In addition, our patient had pulmonary hypertension (PH) that is commonly associated with ACVRL1 mutations, revealing her phenotype was not consistent with isolated ENG genetic mutations. Here, we report a case with hereditary hemorrhagic telangiectasia (HHT) combined with PAH, which is associated with interesting differential diagnosis and etiological analysis. We have discussed the relationship between PH and HHT and the characteristics of PAH in HHT patients.

## Case presentation

A 31-year-old woman complained of “shortness of breath for 2 years with exertion, worsening for 10 months.” Two years ago, in the fifth month of her fourth pregnancy, she suffered exertional dyspnea accompanied by edema of both lower extremities, which was relieved after delivery. In the past 10 months, the patient’s exercise capacity decreased progressively with onset of palpitations, and she had to take rest when climbing to the second floor. She was admitted to a local hospital. The pulmonary systolic pressure was estimated to be 110 mmHg by echocardiography. Computed tomographic pulmonary angiogram (CTPA) showed that the main pulmonary artery was widened, and no filling defect was found. Her symptoms improved with diuretic.

**FIGURE 1:**
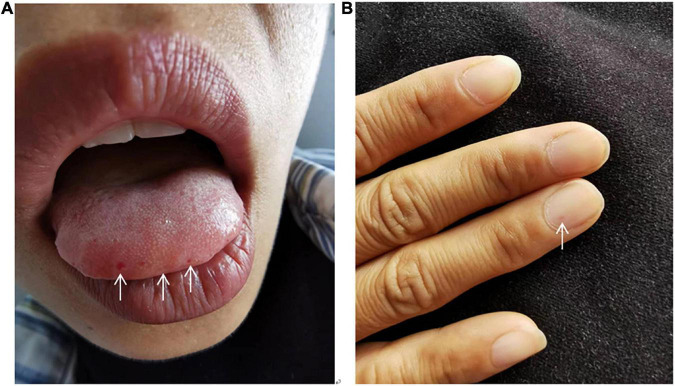
Abnormal inspection. Cyanosis was noted on the lips, and telangiectasias were seen on the oral mucosa **(A)** and fingertips **(B)**.

She had had recurrent epistaxis for more than 10 years, and her eldest and second daughters also had a history of epistaxis. Vital signs revealed a temperature of 36.5°C, heart rate of 85 beats per minute, respiratory rate of 21 breaths per minute, and blood pressure of 119/70 mmHg. Cyanosis was noted on the lips, and telangiectasias were seen on the oral mucosa and fingertips (Figure 1). Auscultation revealed P2 > A2 in the pulmonary auscultation area and grade 3/6 systolic murmurs in tricuspid valve area. No pitting edema of extremities was observed. Arterial blood gas analysis on room air is normal. Fecal occult blood was weakly positive. Complete blood count and decreased ferritin were suggestive of iron deficiency anemia. Liver function revealed slightly elevated bilirubin and brain natriuretic peptide, troponin were normal. Immunoglobulin levels were normal, but with mildly decreased levels of complement3 and complement4. Coagulation function showed reduced activity of protein C. Laboratory data in detail are shown in Table 1.

**TABLE 1:** Laboratory data.

Arterial blood gas analysis	pH 7.45, PCO_2_ 30 mmHg, PO_2_ 95 mmHg, SO_2_ 98%
Complete blood count	hemoglobin 103 g/L, mean corpuscular volume 74.9 fl, mean corpuscular hemoglobin 22.8 pg, mean corpuscular hemoglobin concentration 305 g/L.
Anemia test	ferritin 4.1 ng/ml, normal folic acid, vitamin B12, anti-intrinsic factor antibody.
Liver function	total bilirubin 31.54 umol/L, direct bilirubin 9.32 umol/L.
Cardiac biomarkers	normal
Anticoagulation test	protein C activity 56%, normal activity of protein S and antithrombin-III.
Antinuclear antibody	normal
Rheumatoid antibody	normal
Vasculitis antibody	normal
Tumor markers	normal
Complement	complement3 62.80 mg/dl, complement4 9.25 mg/dl.

Reference ranges are as follows: hemoglobin, 115–150 g/L; mean corpuscular volume, 82–100 fl; mean corpuscular hemoglobin, 27–34 pg; mean corpuscular hemoglobin concentration, 316–354 g/L; ferritin, 11.0–306.8 ng/ml; total bilirubin, 5.00–21.00 umol/L; direct bilirubin, 0–7.00 umol/L; protein C activity, 70–130%; complement3, 70.00–128.00 mg/dl; complement4, 16.00–47.00 mg/dl.

**FIGURE 2:**
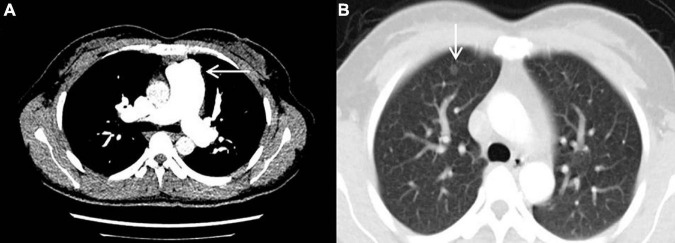
Computed tomography pulmonary angiography. **(A)** No obvious filling defect or vascular malformations were observed. **(B)** The ground-glass opacity that was 7.8 mm × 7.5 mm in diameter and located in the right upper lung lobes was seen.

**FIGURE 3:**
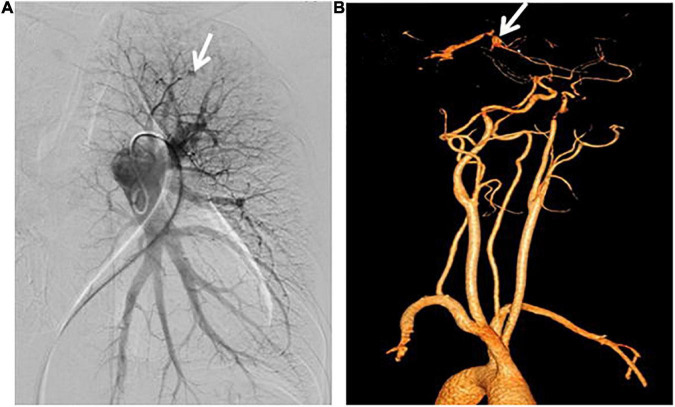
Pulmonary angiography and cranial computed tomographic angiogram. **(A)** Arteriovenous malformations of the left upper pulmonary artery were shown. **(B)** Anterior cerebral artery malformations were revealed.

The result of right heart contrast echocardiography was consistent with pulmonary arteriovenous malformations (AVMs). The result of echocardiography was indicative of pulmonary hypertension (PH). Electronic gastroscopy showed chronic non-atrophic gastritis but no gastric vascular malformations. Pulmonary function showed mild diffusion dysfunction. No obvious filling defect or vascular malformations were observed on CTPA (Figure 2A), but several ground-glass opacities were seen on the lung window of CTPA, where one of 7.8 mm × 7.5 mm in diameter was located in the right upper lung lobes (Figure 2B). The result of right heart catheterization (RHC) is shown in Table 2. Pulmonary angiography showed AVMs of the left upper pulmonary artery (Figure 3A), and anterior cerebral artery malformation was seen in cranial computed tomographic angiogram (Figure 3B). Genetic testing revealed the patient carried heterozygous variants in the ENG c.1195-1196del p.Arg399GlyfsTer2. Except her son, all her three daughters carried a heterozygous variant at the ENG c.1195-1196del locus.

**TABLE 2:** The results of right heart catheter in two admissions.

	First admission	Second admission
Right atrial pressure	5/1 (2) mmHg	6/2 (3) mmHg
Right ventricular pressure	63/0 (21) mmHg	53/–2 (21) mmHg
Pulmonary artery pressure	63/23 (33) mmHg	51/18 (32) mmHg
Pulmonary artery wedge pressure	7 mmHg	10 mmHg
Pulmonary blood volume	5.39 L/min	8.15 L/min
Pulmonary vascular resistance	4.82 Wood units	2.70 Wood units

As a young adult woman, our patient’s main clinical symptom at presentation was exertional dyspnea, and she had recurrent epistaxis, multiple mucocutaneous telangiectasias, and organ involvement. According to Curacao diagnostic criteria (1), together with the results of RHC, the diagnosis of hereditary hemorrhagic telangiectasia (HHT) combined with pulmonary arterial hypertension (PAH) could be made.

She was treated with macitentan 10 mg once daily, furosemide 20 mg once daily, spironolactone 20 mg once daily, and polysaccharide iron complex 300 mg once daily.

Four months later, the patient returned to our hospital. The patient’s exercise tolerance was improved. Repeat RHC revealed in Table 2. Although the mean pulmonary artery pressure (PAP) of the patient did not change significantly, she reported feeling better and meantime pulmonary blood volume and pulmonary vascular resistance (PVR) improved.

## Discussion

Hereditary hemorrhagic telangiectasia is an autosomal dominant inherited disease characterized by multisystemic vascular dysplasia (2). The main genetic mutations are in endoglin (ENG), activin receptor-like kinase1 (ACVRL1), and mothers against decapentaplegic homolog 4 (SMAD4) (3). HHT1 is caused by mutations in ENG gene, while HHT2 is caused by ACVRL1 gene mutations, where TGF-β superfamily signaling pathway has been recognized to play a vital role (4). For our patient, one heterozygous variant was detected in the ENG gene, and the c.1195-1196del variant was located in the ENG gene of exon 9, which was a pathogenic variant according to the American College of Medical Genetics (ACMG) criteria (5). The mutation of ENG c.1195-1196del could cause a frameshift at amino acid 399 of the encoded protein, resulting in an early stop codon at amino acid 400. All types of mutations have been reported, including missense, non-sense, deletions, insertions, and splice site. Most families with HHT have unique mutations, and more than 900 mutations are described (6). According to the analysis of ExAC database and gnomAD database, the population frequency of this variant locus has not been recorded, but this variant was reported in a patient with spontaneous hemothorax (7).

Although PH is increasingly recognized as an important complication of HHT recently (8), whether PH is secondary to HHT or HHT is combined with PH in our patient was required to be discussed. At present, it is believed that the main pathogenesis of HHT-related PH occurs in two ways. First, multiple pulmonary AVMs bring about significant right-to-left shunt, resulting in high hemodynamics and increased cardiac preload. Reverse transmission of pressure leads to PH of a post-capillary subtype. One large study of 175 children confirmed HHT and found that 22% of children with HHT had pulmonary AVMs (9). But PH is not always associated with pulmonary AVMs. Our patient has no obvious hypoxemia and erythrocytosis, which means the right-to-left shunting in pulmonary arteries is not severe. These signs are consistent with the results of pulmonary artery angiography. Second, others may feature significantly increased PAP and PVR with normal pulmonary artery wedge pressure (PAWP), which defines pre-capillary PH, whose histological features are not significantly different from idiopathic pulmonary arterial hypertension (IPAH). A cohort study of 578 HHT patients found that HHT2 patients were more likely to have post-capillary PH than HHT1 patients (10) because such patients often have hepatic AVMs. Trembath et al. found that ACVRL1 gene mutations may lead to the occlusion of pulmonary artery, which results in hereditary PAH (HPAH) and clinical manifestations, such as AVMs (3). Pre-capillary PH in HHT patients is relatively rare and mainly occurs in patients with ACVRL1 gene mutations (4). Therefore, HHT-related PH has primarily been associated with ACVRL1 gene, leading us to wonder whether HHT was the only contributing factor to her PH.

Genetic testing provides clues for a definite diagnosis, but when the patient’s phenotype could not be fully explained by the genotype, it is necessary to review the patient’s medical history and test results to make the best clinical judgment. The etiology can be traced back as follows: (1) Reduced protein C activity and normal activity of protein S and antithrombin-III were found. She did not take warfarin recently, which may interfere with the activity of protein C. The available evidence could not support the diagnosis of hereditary thrombophilia. But she was in a potential hypercoagulable state, which was a risk factor for pulmonary veno-occlusive disease (PVOD)/pulmonary capillary hemangiomatosis (PCH). Although EIF2AK4 gene mutation was not found in our patient, PVOD/PCH could not be completely ruled out due to her manifestations of PAH in the absence of left-sided heart disease, combined with ground-glass nodules and decreased lung diffusion capacity. Unfortunately, high-resolution chest tomography (HRCT) and transbronchial lung biopsy (TBLB) were not underwent. (2) The four times of pregnancies kept the pulmonary blood vessels in a state of higher circulation over an extended period of time. Although the abnormal state was not continuous, the remodeling of the pulmonary capillaries likely could not be recovered. (3) A small amount of right-to-left shunt can be seen in the patient’s right heart contrast echocardiography, but it is currently hard to confirm whether it is a congenital or secondary change. Whether patent foramen ovale worked in the progress of PH is uncertain. (4) Pre-capillary PH could be the result of chronic thromboembolic pulmonary hypertension (CTEPH) (11), but no significant ventilation and blood flow mismatch were observed in ventilation/perfusion scintigraphy, which was vital for the diagnosis of CTEPH. (5). In addition, chronic anemia caused by epistaxis led to increased cardiac output and may also have contributed to the development of PH.

Although PAH in HHT patients with ENG gene mutation is not first reported, the diagnosis and etiological analysis in this group is still challenging. On the one hand, hereditary PAH in patients with pulmonary AVMs in combination with HHT is difficult to be distinguished clinically from IPAH, as it is usually associated with dysfunction of ALK-1, the product of ACVRL1 gene (12). Some studies found approximately 70% of cases with HPAH are associated with bone morphogenetic protein receptor type II (BMPR2) gene mutation and less than 1% of patients with HHT suffered HPAH caused by a mutation in the ACVRL1 gene (8, 13). However, HPAH patients with ACVRL1 gene mutations are frequently asymptomatic and not combined with HHT (14). Besides, several less common HHT gene mutations were identified in HPAH patients, such as ENG gene and growth differentiation factor 2 (GDF2) gene (15). On the other hand, as a rare complication of HHT, PAH could be likely related to high-output heart failure. A case of 55-year-old woman with HHT combined with high-output heart failure was reported, and her PVR was elevated to 5.5 Woods units prior to the treatment (16). Compared with relatively temporary high circulation state of our patient, she was in a persistent state due to the presence of hepatic AVMs. Besides, a case of 26-year-old woman with HHT with pre-capillary PH was reported. After she underwent the therapy of pre-capillary PH, high cardiac output and PAWP were surprisingly unveiled (17), which suggested that high cardiac-output state could be masked.

In summary, PH could result from multiple different issues and is likely multifactorial. Standardized diagnosis, seeking causes, and early identification of risk factors are essential to improve the prognosis of patients. Additional causes should be suspected and sought out when unusual clinical phenomena occur. As a rare disease, if HHT was combined with PH, it could easily be diagnosed as idiopathic PAH or attributed to HHT alone if one is not mindful of other potential contributions. Therefore, it is vital to evaluate the underlying reversible causes.

## Limitations

This was a retrospective study and the lack of information was the shortcoming of our study. Up to the time we chose this case, the patient had been hospitalized twice and we read all the data in the electronic medical record, but the patient did not complete all important examinations, including brain magnetic resonance imaging, HRCT, and TBLB.

## Future directions

In patients with HHT, the pathogenesis of PH is complex and multifactorial and the phenotypes are diverse. It is not clear how to identify whether PH is secondary to HHT or HHT is combined with PH accurately. It is difficult to tell the differences between HHT-related PAH and PAH in the general population. Further researches are required to explore the pathogenesis of PH in HHT patients.

## Conclusion

We report such an ENG-related case of HHT combined with PAH in a young adult woman with minor pulmonary AVMs for the first time. We found that even if high-output heart failure was not continuous in HHT patients, irreversible pulmonary vascular remodeling might occur and pre-capillary PH was caused.

## Data availability statement

The original contributions presented in this study are included in the article/supplementary material, further inquiries can be directed to the corresponding author/s.

## Ethics statement

Written informed consent was obtained from the individual(s) for the publication of any potentially identifiable images or data included in this article.

## Author contributions

DL collected the patient’s information, summarized the literature data, and wrote the manuscript. FX collected the patient’s information and wrote the manuscript. QG and ZZ were the major contributors in revising the manuscript. All authors read and approved the final manuscript.

## Abbreviations

ACMG: American College of Medical Genetics
AVMs: arteriovenous malformations
BMPR2: bone morphogenetic protein receptor type II
CTEPH: chronic thromboembolic pulmonary hypertension
CTPA: computed tomographic pulmonary angiogram
HHT: hereditary hemorrhagic telangiectasia
HPAH: hereditary pulmonary arterial hypertension
HRCT: high resolution chest tomography
IPAH: idiopathic pulmonary arterial hypertension
PAP: pulmonary artery pressure
PAH: pulmonary arterial hypertension
PAWP: pulmonary artery wedge pressure
PCH: pulmonary capillary hemangiomatosis
PH: pulmonary hypertension
PVOD: pulmonary veno-occlusive disease
PVR: pulmonary vascular resistance
TBLB: transbronchial lung biopsy
RHC: right heart catheterization.
